# Programmable Multiwavelength Radio Frequency Spectrometry of Chemophysical Environments through an Adaptable Network of Flexible and Environmentally Responsive, Passive Wireless Elements

**DOI:** 10.1002/smsc.202200013

**Published:** 2022-03-27

**Authors:** Manik Dautta, Amirhossein Hajiaghajani, Fan Ye, Alberto Ranier Escobar, Abel Jimenez, Kazi Khurshidi Haque Dia, Peter Tseng

**Affiliations:** ^1^ Department of Electrical Engineering and Computer Science University of California Irvine Engineering Hall #3110 Irvine CA 92697 USA; ^2^ Department of Biomedical Engineering University of California Irvine Engineering Hall #3110 Irvine CA 92697 USA; ^3^ Present address: Department of Electrical Engineering and Computer Science University of California Berkeley Cory Hall #550 Berkeley CA 94720 USA

**Keywords:** multiparametric readout, radio frequency (RF) sensors, sensor networks, spectroscopy, wireless sensing

## Abstract

Readout of multiparametric environmental signals typically uses discrete sensing formats that individually require unique signal conditioning circuitry and/or processing pathways. Here, adaptable sensor networks composed exclusively of passive material architectures that enable spectrometric comonitoring of chemical or physical environmental signals are proposed. Herein, a single radio frequency (RF) reader wirelessly interacts first with an intermediate wireless relay coil—this is tunable in length and can be designed to conform around surfaces. This relay (that is fused on textiles or surfaces) is then wirelessly coupled to arrays of passive RF sensors with individually programmable flexibility/reactivity to environmental signals. Multiple chemical and physical signals can then be monitored within the single spectral readout of a wearable reader. This technique can probe over tunable length scales, and is robust to mechanical disturbances that limit present techniques. As a proof of concept, this approach is used to comonitor chemophysical metrics such as nutrients, temperature, pressure, pH, and more on the skin or in utensils with a single readout. This technique may form a cornerstone of zero‐microelectronic sensor networks.

## Graphical abstract

Complex, multiparametric signals can be measured in a single spectral readout via an adaptable network of fully passive (and zero‐microelectronic) elements—such networks conform and flex along textiles or surfaces while enabling long distance and mechanically stable measurement of the chemophysical environment.

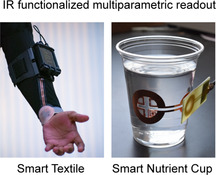

## 1 Introduction

Spectrometric readout has wide uses in modern science, where electromagnetic (EM) radiation of varying frequencies can be used to probe matter in its varying forms (whether simple or complex). Spectral signatures that manifest from such EM interactions are used as an important identification tool in chemistry, physics, and biomedicine.^[^ ^1^, ^2^, ^3^, ^4^ ^]^ A major characteristic of this readout is its data‐rich nature, which enables a significant amount of information to be extracted in a single measurement. This manifests from the differential response of matter to varying EM wavelengths that may span X‐rays, UV–vis, terahertz, and radio frequency (RF).^[^ ^5^, ^6^, ^7^, ^8^ ^]^ Some of the most widely utilized methods include a material‐under‐test that is excited directly by a broad spectrum of radiation—examples include Fourier transform infrared/UV–vis^[^ ^9^, ^10^ ^]^ and mass spectrometry,^[^ ^11^, ^12^, ^13^ ^]^ wherein characteristic peaks over a broad spectrum yield rich, multiparametric data on the material‐under‐test.

Modern processing techniques enable a more focused cousin of this approach, wherein matter is organized into structures that interact with radiation in a directed way, such as to exhibit resonance phenomena.^[^ ^14^, ^15^, ^16^, ^17^ ^]^ This approach can be used to create more selective/sensitive sensors that are typically intended to measure a single value.^[^ ^18^, ^19^, ^20^, ^21^ ^]^ A common example of this is RF sensors, wherein conductive traces are patterned so as to resonate when excited by RF waves.^[^ ^22^, ^23^, ^24^, ^25^ ^]^ Such an approach has been adapted to build sensors and biosensors sensitive to a variety of chemophysical signals, such as pressure,^[^ ^26^, ^27^ ^]^ temperature,^[^ ^28^, ^29^, ^30^ ^]^ glucose,^[^ ^31^, ^32^ ^]^ salinity,^[^ ^33^, ^34^ ^]^ nutrients,^[^ ^35^, ^36^ ^]^ and more. Despite the emerging versatility of this approach, RF sensor readout is still highly limited, as typically only a single sensor is assessed at a time, and the technique is not stable to mechanical noise because readout coil and sensor alignment are typically not fixed.

Here, we study a form of programmable RF spectrometry, wherein a single readout of RF spectra can be used to assess a wide variety of desired chemophysical signals from the environment. This is in contrast to standard readout of multiparametric signals where individual sensing formats require unique signal conditioning circuitry and/or processing. Here, RF waves interact with multilayers of electronics‐free patterned, wirelessly coupled elements that can be engineered to various length scales, to deform or attach around surfaces, and tuned to controlled reactivity to chemical or physical signals. This is broad expansion of more basic iterations of this technique that monitors arrays of pressure sensors via planar readout coils,^[^ ^24^, ^37^ ^]^ or the radiation of an array of temperature sensors.^[^ ^38^, ^39^ ^]^ In our study, RF signal is first mediated by passive intermediate relay coils that are wireless and electrically disconnected from other elements. This can transfer signal over intermediate distances, and can be fused onto textiles^[^ ^40^, ^41^, ^42^ ^]^ or conform over surfaces.^[^ ^43^ ^]^ These relays are then wirelessly coupled to RF sensors with tunable environmental reactivity—demonstrated herein include pressure, temperature, salinity, and nutrients (sugars/salts/fats). This then forms a multiparametric network composed exclusively of passive material architectures. Beyond the fully passive/wireless coreadout of multiparametric signals, this approach is significantly more robust in comparison to traditional RF readout—this is because intermediate coil to RF sensor alignment can readily remain fixed through design. In general, any capacitive or resistive sensor type may be integrated with our technique, as these readily build into RF sensors such as those we show herein. As proof‐of‐concepts, we demonstrate multiparametric, chemophysical readout from wireless wristbands and SmartCups that are infused with multilayers of interacting, flexible/reactive wireless elements.

## 2 Results and Discussions

**Figure 1:**
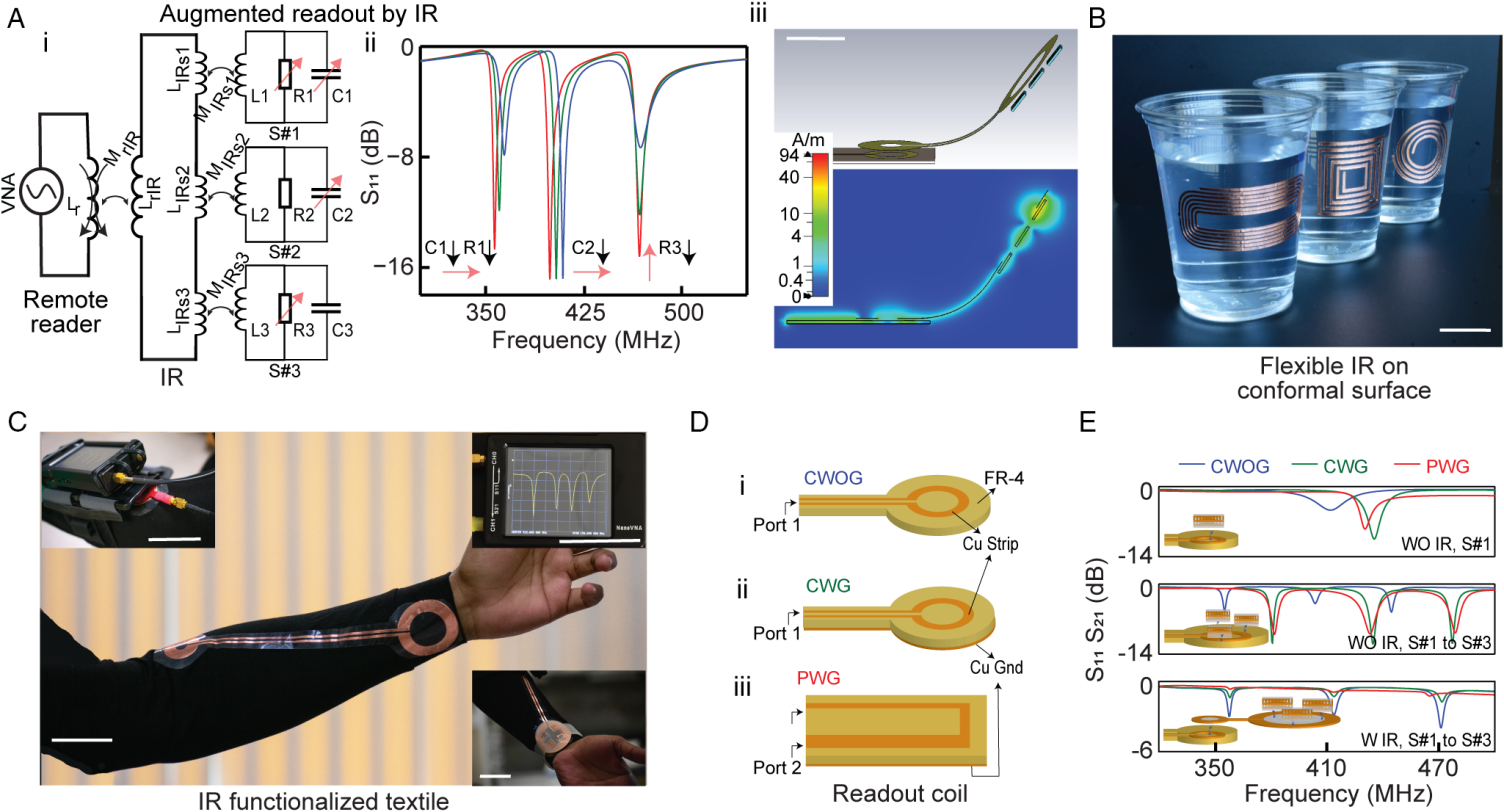
Programmable multiwavelength RF spectrometry of the chemophysical environment. A) A network composed of multilayers of passive (zero‐electronic) elements enables single readout comonitoring of complex signals. (i) Circuit diagram of reader wirelessly coupled to an IR, in turn wirelessly coupled to tunable RF sensors, (ii) RF simulation of the spectral readout where sensor 1 is perturbed by both R1 and C1, sensor 2 is perturbed only in C2, and sensor 3 is perturbed only in R3, (iii) geometry used in finite element method (top) and corresponding magnetic field distribution showing magnetic coupling between elements. B) Flexible IR integrated on the outer surface of cups. C) IR‐integrated smart textile to facilitate multiparametric wristband readout. Insets are the placement of the wristband and direct readout from wearable NanoVNA. D) Readout antenna structures studied herein (i) circular without ground (CWOG), (ii) circular with ground (CWG), and (iii) planar with ground (PWG). E) Spectral readout from various network configurations: single sensor without IR (diameter of the readout coil loop is 25 mm), multiple sensors without IR (diameter of the readout coil loop is 50 mm), and multiple sensors with IR (diameter of the IR loop is 50 mm). The standard vertical distance between readout coil/IR loop and sensors is 0.5 mm. For multisensor readout, the orientation of the sensors remains constant. Scale bars are 5 cm.

Our approach is composed of three types of RF elements that are wirelessly coupled to form the complete circuit, as shown in **Figure** **1**Ai. First are readout coils that form the initial inductive link into our passive sensor network, and that is probed via direct wired connection to a reader (such readers include tabletop or wearable vector network analyzer (VNA)). The inductive readout coil can be designed as a one port circular coil for S_11_ or a two port microstrip patch line for S_21_ spectral response readout (Figure S1, Supporting Information). Herein, we utilized either a 25 mm diameter circular readout coil (feed line length 35 mm) or U‐shaped microstrip patch line (25 and 35 mm traces) that are selectively integrated with FR‐4 substrate (fabrication of which is discussed in Supporting Information). Second is an intermediate relay (IR) coil that is untethered from all other elements. This is wirelessly coupled with the readout coil, transferring the EM fields to subsequent sensors along its pathlength or through designed inductive terminals. This IR plays an important role in the structure—in our manifestation it is synthesized on flexible substrate, and subsequently fused onto curved surfaces or textiles. This allows RF signal to transmit over materials/substrates relevant to our daily life, and can be tuned to transfer signal over arbitrary distances. Beyond facilitating information from localized sensing nodes, these enhance the mechanical robustness of the sensing network. Sensor alignment to intermediate coil is simple to maintain due to the flexible/routing nature of the IR—as will be seen this helps stabilize the spectral readout to misalignment between the readout coil and network. This adds significant flexibility to the final passive sensor network. We demonstrate various practical manifestations wherein the IR coil is embedded alongside a cup to enable a SmartCup for comonitoring nutrients in food (Figure 1B), or fused on a textile to facilitate readout of a wristband from across the arm (Figure 1C, and S2, Supporting Information).

Third and lastly are passive and wireless RF sensors with individually tunable mechanical or chemical reactivity. These sensors are passive resistance–inductance–capacitance structures that are built to modulate with environmental signals. Herein, we utilize broadside coupled, split ring resonating architectures that have been previously characterized by our group. One key aspect of our strategy is the utilization of interlayer‐RF sensor design schemes such as those we have previously demonstrated.^[^ ^30^, ^32^, ^35^ ^]^ Modulation of the lumped resistance of a sensor changes the magnitude, while modulation of the lumped capacitance shifts the resonant frequency of its spectral response. Individual sensors are built with specialized materials (both within and around the sensing architecture) and thus rendered selectively sensitive to metrics such as glucose, sugars, salts, fats, pressure, temperature, and more.^[^ ^25^, ^30^, ^32^, ^35^, ^44^ ^]^ Importantly, these structures are readily tuned to respond/resonate at different wavelengths, and thus occupy individual frequency bands during spectral readout. This occurs by simply varying the thickness of the interlayer. This allows us to readily tune any sensor of a set square area (size footprint) to hit variable operating frequencies. Thus, for our sensors (0.5–1 cm wide) we could readily tune response to occupy various desired bands for different environmental responses. These sensors are oriented along the IR coil, and whose resonance can be probed through the intermediate relay signal.

The final, versatile structure is a fully passive sensor network (requiring zero electronics) that can monitor complex chemophysical signals in a single readout. Figure 1Aii shows the RF simulation of coreadout of three sensors (numbered S#1, S#2, and S#3, respectively). Here, both C1 and R1 of S#1, only C2 of S#2, and only R3 of S#3 are perturbed. Enlargement of R3 decreases the signal magnitude, reduction in C2 increases the resonant frequency, while reduction in both R1 and C1 decreases the signal magnitude and increases the resonant frequency, respectively. These modulations map exclusively to the spectral band occupied by individual sensors. Figure 1Aiii shows a finite element simulation of the magnetic fields within a sample network. This field distribution displays the multiple layers of wireless magnetic coupling between readout coil and sensors via the IR. This system exhibits additional power loss in comparison to traditional RF sensor readout due to the additional wireless couplings—specifically the coupling between readout coil and IR, and coupling between IR and sensors. The effect of this interceding coil can be seen in the reduced magnitude S_11_ response of the multicoil network as opposed to the direct readout of sensors (this is for the same input dBm to both configurations). The impact of the lower S_11_ is that shifts in the magnitude and frequency of resonant sensors may become more difficult to resolve. A higher power may be used to increase the total S_11_ response, and thus improve the readout of very low‐sensitivity sensors, but there is an upper limit to the total power that may be applied in wearable, or close‐to‐body applications. Thus, in networks using an IR require moderate‐to‐high sensitivity sensors are be required in near‐body environments. However, we note that this type of moderate‐to‐high sensitivity is not difficult, as all our demonstrated sensors herein are easily probed/measured with −5 dBm (≈300 mW), which is standard for many wearable applications in nearfield.

In this article, we studied three readout antennas for targeting different applications: circular without ground plane (CWOG), circular with ground plane (CWG), and patch with ground plane (PWG), as shown in Figure 1D. The CWOG is a circular loop readout coil pasted on FR‐4 substrate which has one port connected to the VNA, whereas the CWG is the same readout coil but the other side of FR‐4 substrate has a conductive ground plane. PWG is a microstrip patch line which has two ports connected to the VNA, and the common ground pin is shorted via the connection with the ground plane on the other side of the FR‐4 substrate. A detailed layout is presented in Figure S1, Supporting Information. Sensors are of variations of interlayer RF structures, but our fundamental structure was a 15 mm‐wide, 3.25 turn spiral square trilayer structure (Figure S3, Supporting Information). This structure is modulated in several ways to broadly tune the sensor to different resonant frequencies while retaining the same footprint: via modification of the coil turn number or interlayer thickness. Figure 1E compares the spectral readout of single and multiple sensors when probed by various readout antennas, with and without an IR interceded within the structure. We additionally studied the effect of different vertical distances between the antenna and sensors, which modulates the spectral response due to changing coupling coefficient (Figure S4, Supporting Information). Finite‐difference time‐domain simulation was additionally performed to model the behavior of the sensor and readout coil resonant spectra, and to map the EM field distribution (Figure S5, Supporting Information). It can be seen that the grounded structures exhibit a larger EM field close to the readout coil; however, this decays more rapidly than the ungrounded structure as we move away from the coil. Both **E** and **H** fields are higher with CWG than CWOG at 3 mm separation between the readout coil and sensor—this matches the higher *Q* measured with CWG. We additionally simulated the effect of bending on sensor readout (Figure S6, Supporting Information). As shown in the following figure, the impact of one or two large folds is a minor shift in the measured resonant frequency/magnitude of the sensor. This shift is around ±0.7 MHz (0.2% shift) in frequency and 2 dB in magnitude. This puts a limitation on the sensitivity of our sensors in the case of dynamic bending environments, which must possess a sensitivity higher than this “noise” in order to be measured properly. Figure 1E (middle) shows the coreadout of three sensors each tuned to different resonant frequencies. Interestingly, the CWOG coil structure exhibits a higher amplitude than the grounded structures in the presence of an IR (Figure 1E bottom). The slower decay of EM field away from the ungrounded structure improves signal transmission through this intermediate structure, which must be wirelessly coupled to over a set distance. This knowledge can be utilized to optimize network readout and design depending on the presence of an IR, and the coupling distance of the various elements of the network. This will be seen in the measurement/implementation of wireless wristbands and “smart” cups later in this study. As an additional note, in all such scenarios the EM field is seen to be strongly confined between individual sensor and the readout coil, which means there is negligible magnetic cross‐coupling among nearby sensors.

**Figure 2:**
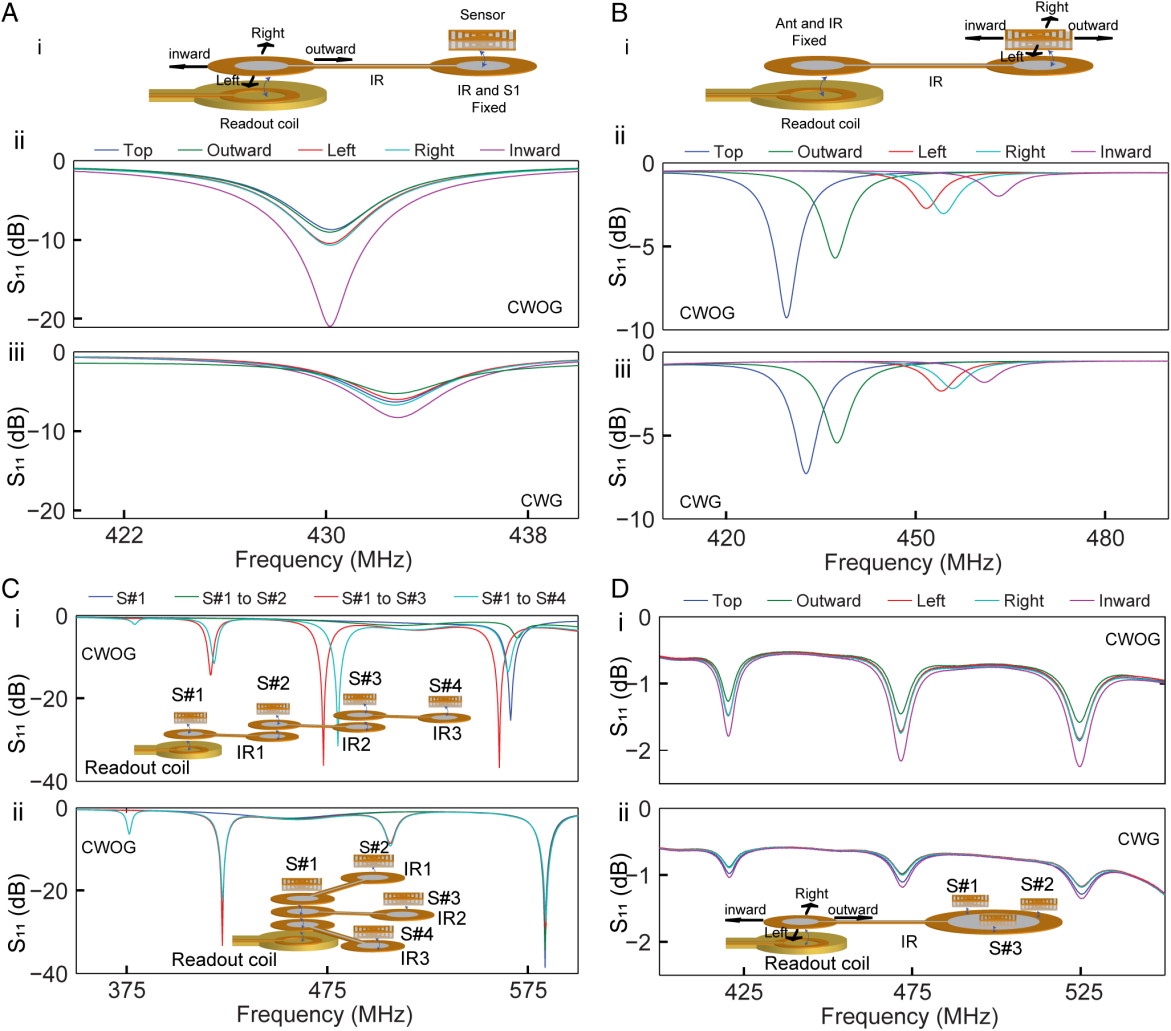
Augmented readout via network coupling through an IR. A) Effect of the alignment between readout antenna and IR when IR and sensor orientation is fixed: (i) schematic presentation of the orientation of the antenna, IR, and sensor, network S_11_ response by (ii) CWOG, and (iii) CWG. B) Effect of the alignment between IR and sensor while antenna and IR are fixed: (i) schematic of network orientation, network S_11_ response by (ii) CWOG and (iii) CWG. C) Readout of multiple sensors through multiple IR. Network S_11_ response during (i) series and (ii) parallel extension. Insets are the network orientation. D) Readout of multiple sensors by IR. Effect of the alignment between readout antenna and IR when IR and sensor orientations are fixed: network S_11_ response by (i) CWOG (ii) CWG. Inset is the network orientation.

We next explored various network orientations involving the presence of the IR. First, we studied the effect of the alignment of the readout antenna and an IR30/30 (30 mm loop diameter for both readout coil and sensor coupling) while the IR and sensor placement is fixed (**Figure** **2**A, exploded view of the schematic in Figure S7, Supporting Information). As can be seen, the translational alignment between antenna and the IR has little to no effect on the resonant frequency. This stability in the spectral response importantly means that sensors that exhibit shifts in resonant frequency due to environmental perturbations remain measurable even if the readout coil is misaligned from the sensor network. This enhanced mechanical stability is important because this readout coil to network alignment is often not fixed because the reader is commonly brought up to the network and subsequently removed after readout. Note that sensors that shift in magnitude are still measurable given their sensitivity is larger than the magnitude shifts induced by perturbation (this can be tuned by targeting less sensitive regions to align/realign the readout coil to the network). Next, we fixed the antenna and IR30/30 placement, and studied the effect of the IR and sensor alignment (Figure 2B). As expected, we observed that sensor coupling is strongly dependent on the orientation of the sensor with the IR, which can result in shifts to both the resonant frequency and the signal amplitude. This means that this IR–sensor alignment should remain fixed throughout measurement. We note that this is the primary purpose of the IR, which is flexible/conformable and can permanently route signal to desired regions as required by application. This instability is similar to when the readout coil and sensors exhibit mechanical translations without the presence of an IR (Figure S8, Supporting Information). Multiple IR may additionally be coupled to the readout coil via series or parallel extension (Figure 2C), where differing number of sensors are added to the network. In series extension, sensors placed further in the network modulate the spectral response due to sensor cross‐coupling. This disappears for parallel extension, where additional sensors can be added without modulating the measured resonant frequency of previous sensors (in this scenario sensors are coupled only to the IR but not each other). Multisensor networks are additionally stable to mechanical translation (Figure 2D). These results broadly indicate that IR interceded sensor networks can provide stabilized readout given a wide variety of scenarios. One limitation of using the IR is elongating the ends will lead to a reduction in signal amplitude (and thus limits the practical sensitivity of the measurement). There is a very direct trade‐off, where very long distances will require either higher sensitivity sensors, or higher input power in order to resolve measurements.

We performed additional simulations to illustrate the effect of increased length on the measured S_11_ (Figure S9, Supporting Information). At 50 cm the measured S_11_ of sensors does decrease in comparison to shorter distances, as various sensors will exhibit magnitude shifts of 1–4 dB at this distance (higher frequency sensors are more robust to increased distances). From these findings it appears as though at very long distances sensors can be pushed to higher operating frequencies to maintain similar readout sensitivity. In general, we have found even −1 dB of amplitude response to be sufficient for proper measurement of our particular RF sensors.^[^ ^36^ ^]^

**Figure 3:**
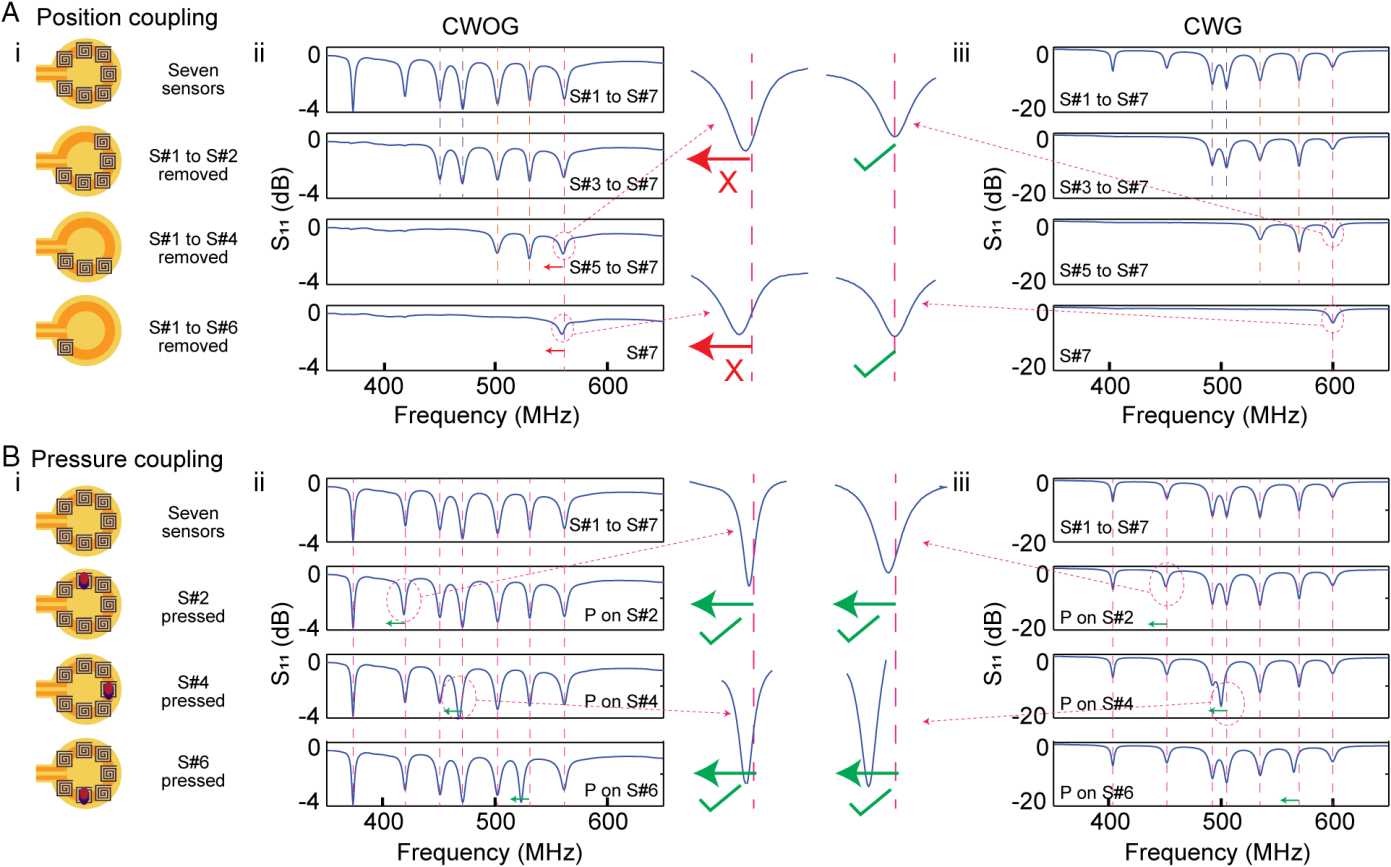
Cross‐coupling effect among sensors. A) Effect of positional coupling between sensors: (i) schematic presentation of sensor array orientation during experiment, and S_11_ response from sensors with (ii) CWOG and (iii) CWG readout antennas. In between is a blow‐up plot highlighting the shift or lack of shift in resonant frequency due to coupling. B) Effect of sensimetric coupling between sensors (applied pressure): (i) schematic presentation of applying stimuli to various sensors, and S_11_ response from sensors with (ii) CWOG and (iii) CWG readout antennas. In between is a blow‐up plot highlighting the shift in resonant frequency of the spectral peak linked to respective sensors. No other peak exhibits a shift.

In such a passive network, a large number of sensors can potentially be accommodated, the limits of which can be assessed through measurement of the cross‐coupling among sensors. We initially tested sensor positional coupling as they were arrayed in increasing numbers above readout coils (**Figure** **3**A). This type of coupling is not as important as sensor measurement coupling, the results of which will follow. Seven sensors were placed initially on the readout coil (the minimum physical distance of the adjacent sensors is 2 mm) and sensors were removed one by one. We found that for ungrounded readout coils, removal of sensors from a dense network could lead to small shifts in the measured resonant frequency of remaining sensors on the network (Figure 3Aii). On the other hand, for CWG, there is no effect due to removal of sensors from seven to one, as shown in Figure 3Aiii (full data on these experiments is shown in Figure S10, Supporting Information, for CWOG and in Figure S11 for CWG, Supporting Information). In addition to as shown earlier, the measured sensor amplitude is stronger for CWG during this direct multisensor readout. This type of coupling effect due to sensor placement was additionally tested with an IR (Figure S12, Supporting Information), and shows a positional effect for both CWOG and CWG in agreement with this observed effect. This implies that given a dynamic sensor network wherein sensors may be picked‐and‐placed, grounded RF elements will simplify sensor measurement due to minimal positional coupling. One fundamental limitation of such system is the maximum number of sensors that can be measured. For low‐cost (wearable) VNA systems that accurately measure response up to ≈1.5 GHz, the primary limitation comes in the bandwidth that sensors occupy. Generally, approximately 100 MHz band per sensor is more than sufficient to properly assay individual sensors (smaller bandwidth is required for more sensors that shift less in frequency with perturbation). For low‐cost systems, with sensors that occupy 100 MHz, we can assume that we can accommodate around 15 RF sensors.

More important is assessing how the perturbation of single RF sensor may impact the total spectral response of the network (Figure 3B). Seven pressure sensors were placed on the readout coil and each sensor perturbed in sequence (Figure S13 for CWOG and S14 for CWG, Supporting Information). In these mechanical sensors, the resonant frequency will shift due to an applied mechanical pressure. For such a static network, for CWOG, CWG, and PWG we found no disturbance/cross‐coupling in the total spectra of the network due to the perturbation in individual sensors (Figure 3Bii,iii, PWG shown in Figure S15 and S16, Supporting Information). Additionally, no sensimetric coupling is observed with an additional interceding IR element (Figure S17, Supporting Information). In conjunction with measurements on sensor positional coupling, this data suggests that the presence of individual sensors may modulate the induced EM field around the readout coil with ungrounded readout (thus perturbing measurements if sensors are removed), however individual sensor response does not directly cross‐couple to the total network. Importantly, this implies that with a static and defined network, given any measurement modality used or with/without the presence of an IR, individual sensor response links exclusively to its designated wavelength. As will be seen, such static networks can readily be engineered by embedding coils and sensors along structures with our flexible fabrication protocols.

**Figure 4:**
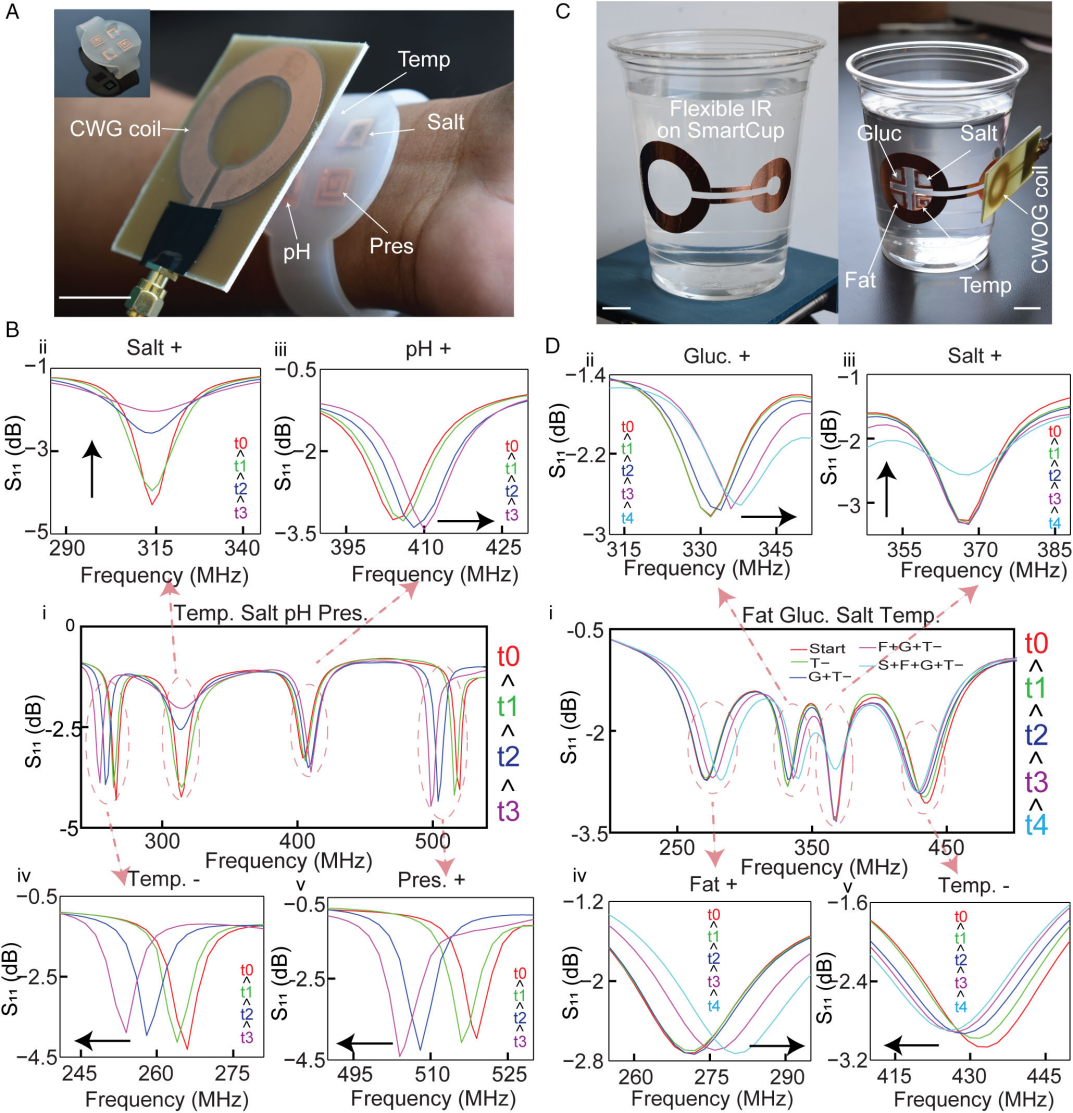
Functional systems for monitoring chemophysical state. A) Multiparametric readout from a wearable wristband. B) Spectrometric coreadout of sensor state: (i) evolution of spectra after completed perturbations, and (ii‐v) zoom‐in of network S_11_ response due to modulating salt (0–10 mg dL^−1^), pH (4–7.4), temperature (40 °C to room), and pressure (manual). C) A Smartcup for comonitoring nutrients in a drink. D) Spectrometric coreadout of sensors state: (i) evolution of spectra after completed perturbations, and (ii‐v) zoomed network S_11_ response of the sugar‐optimized (0–100 g L^−1^), salt‐optimized (0–25 mg dL^−1^), fat‐optimized (0–20 μL), and temperature (50 °C to room) sensors after completed perturbations. Scale bars are 2 cm.

We implemented such studied passive wireless networks to monitor the chemophysical state of objects and environments relevant to our daily life. First was with a wristband with four sensors that enable coreadout of salt, pH, temperature, and pressure simultaneously (sensor structures are shown in Figure S18, Supporting Information). The sensing characteristics of such sensors are presented in a previous study.^[^ ^29^, ^30^ ^]^ In general, capacitive‐based sensors shift up to 20% in resonant frequency with varying input, while loss‐based sensors will modulate up to 80% in magnitude. In this wristband (**Figure** **4**A), temperature and pressure sensors are completely sealed within the silicone; however, salt and pH sensors have a bottom side opening to enable access to the sweat. As demonstrated previously, such passive sensors can individually be readout wirelessly without any microelectronics at the sensing node. Such sensors can be comonitored with an intermediate relay fused on textile (Figure 1B), or directly with the readout as shown in Figure 4A. As CWG elicits a higher magnitude response if there is no IR, we used a 5 cm CWG antenna to coread sensor response simultaneously through direct readout. We tested the ability of our sensors to monitor analytical‐to‐physical signals around human subjects (Figure 4B). Typical probing power for VNA (wearable and otherwise) maxes out at around −6 to −5 dBm (≈300 mW). This is below near field communication power standards (≈1 W), which have a measured specific absorption rate of over an order of magnitude below upper limits for the human body.^[^ ^45^ ^]^ We estimate that the allowed maximum power would be a bit over an order of magnitude greater than our current VNA (around the power limit utilized in most Qi chargers, 10 W). This type of excess power is unnecessary if sensors are well designed around bodily stimuli. Figure 4Bi shows the original recorded spectra and modified spectra, where individual ii) salt, iii) pH, iv) temperature, and v) pressure sensor response is shown a larger view. The stimuli were generated individually as follows (to validate the lack of cross‐coupling among sensors): the temperature sensor was heated by the hot air flow, pressure sensor was mechanically stimulated by various weights, NaCl was added to the salt sensors, while deionised (DI) water was added to the pH sensor. As expected, the resonant frequency of the temperature sensor decreases while cooling as the permittivity of the PEG‐1500 interlayer material increases at lower temperature. Resonant frequency of the pressure sensor decreases with pressure as pressure decreases the interlayer thickness. The magnitude of the signal of the salt sensor decreases as salt penetrates and increases the conductivity of the interlayer PEGDA700 hydrogel. The resonant frequency of the pH sensor increases with the DI water (pH ≈ 7), as the *p*(NIPAM*‐co*‐AA) swells from pH 4 to pH 7. Such a wearable wristband enables a passive and wireless multiparamatric readout of the bodily state without any electronics required on the body.

To characterize a functional network containing an IR, we developed a Smartcup that is integrated with our recently developed novel biosensors (sensor structures shown in Figure S19, Supporting Information) for the discrimination and coreadout of nutrients direct from food.^[^ ^35^ ^]^ In the previous study, we utilized multiscale engineering of silk biopolymer‐interlayer constructs to synthesize different sensors tuned to directly measure salts, sugars, and fat content from food. A major advantage of the spectral approach demonstrated herein is that we can measure our varying optimized nutrient sensors simultaneously, easing the coreadout of multiple nutrients in complex inputs. We additionally utilized a temperature sensor alongside three optimized nutrient sensors (tuned to salt, sugar, fat) in the inner side of the Smartcup. These sensors were carefully aligned to an IR that was fixed on the outer side of the smart cup. This forms a stable, passive wireless network with zero‐electronics that is affixed on a cup. As CWOG elicits a higher magnitude response from the network if an IR is used, we used a 2.5 cm CWOG antenna with the IR to coreadout the sensors response simultaneously shown in Figure 4C (left: placement of a flexible IR on the Smartcup, right: placement of the sensor, IR, and antenna). We performed testing of the nutrient monitoring from the Smartcup, which reports on temperature, salt, sugar, and fat (Figure 4D). Figure 4D i) is the original recorded signal and modulated response, where ii) glucose, iii) salt, iv) fat, and v) temperature sensor temporal response is each highlighted in a larger view. These sensors have previously been validated to measure nutrient content while directly exposed to foods (teas, meat, milk, etc.); however, they do exhibit sensimetric cross‐coupling in nutrient response because they are partially selective (this is decoupled using postprocessing analysis). Here, to properly validate that individual sensors do not cross‐couple to the full spectra of network each biosensor is probed in a mini‐well through individual perturbation of their respective target nutrient. In addition, all sensors exhibit a response time which must be monitored. The temperature sensor was heated to 50 °C and let it cool in a 40 °C environment, validating the temperature sensor response does not elicit a change in the readout of other sensors. Glucose was then added to the sugar biosensor, and this increases the resonant frequency due to biopolymer swelling. At the same time, the temperature sensor is still modulating to a lower frequency because of residual lag in the temperature sensor response; however, the remaining sensors still do not exhibit any change as they have not undergone perturbation. Next, oleic acid is added to the fat sensor, where replacement of high permittivity water with low permittivity oleic acid reduces the capacitance of the structure. Now all the sensors except the salt‐optimized biosensor are exhibiting expected temporal shifts in accordance with the characteristics of the individual sensor. Finally, we added NaCl to the salt sensor, which increases the conductivity of the interlayer silk and reduces the signal Q/magnitude. The complete spectra of the Smartcup stabilize to its final state in accordance with the final state of each individual temperature or nutrient sensor. This validates the measurement capabilities of flexible/reactive passive network in a practical setting.

## 3 Conclusion

We have demonstrated adaptable, passive wireless sensor networks composed exclusively of material architectures without any electronic components. Here, intermediate relays allow signal to transmit across longer distances and over curved surfaces, while individually placed passive wireless sensors along the network enable the comonitoring of chemical and physical signals. Such a strategy resolves many traditional issues hampering both electronically mediated and passive wireless sensor readout. A single readout enables complex multiparametric signal extraction without any unique circuitry. Additionally, this network readout is robust to mechanical perturbation (a major issue with standard readout), and the IR allows the network to span across unique environments such as the body or utensils. Our fabrication techniques allow the integration of network components into a multitude of environments, such as textiles, curved surfaces, and more. Such strategies may become the cornerstone of next‐generation sensor networks that require no microelectronic components.

## 4 Experimental Section

The experimental details are provided in the Supporting Information.

## Conflict of Interest

The authors declare no conflict of interest.

## Author Contributions

M.D. and P.T. conceptualized the project. M.D. performed the experiments with assistance from A.H., A.E., A.J., and K.D. The simulations were done by F.Y. All the authors discussed the results. M.D. and P.T. wrote the article. Experiments involving human subjects were performed with informed consent under protocol HS#2018‐4843 from the UCI Institutional Review Board.

## Supporting information

Supplementary Material

## Data Availability

The data that support the findings of this study are available from the corresponding author upon reasonable request.
