# Supplementary material for: Programmable Multiwavelength Radio Frequency Spectrometry of Chemophysical Environments through an Adaptable Network of Flexible and Environmentally Responsive, Passive Wireless Elements
Source: Small Sci. 2022 Mar 27;2(6):2200013. doi: 10.1002/smsc.202200013 (PMC11936011; doi:10.1002/smsc.202200013)
Supplement: Supplementary file 1 — Supplementary Material [file SMSC-2-2200013-s001.pdf]

Supplemental Information

Title: Programmable multi-wavelength RF spectrometry of chemophysical environments through and adaptable network of flexible and environmentally-responsive, passive wireless elements.

Manik Dautta[1], Amirhossein Hajiaghajani[1], Fan Ye[1], Alberto Ranier Escobar[2], Abel Jimenez[1], Kazi Khurshidi Haque Dia[1], and Peter Tseng[1,2,]*

Experimental Design:

Metal Pattern Fabrication: Metal patterns were designed using 2D design tools (such as Layout Editor), and an electronic cutter (Silhouette Cameo 4) was used to create patterns by cutting a conductive foil. The negative pattern of the metal features was removed via a tweezer and then transferred to different substrates.

Readout Antenna and IR Fabrication: For readout antenna and IR, copper foil was used as the conductor with adhesive on the back protected by a glossy paper. After fabrication, the antenna was transferred to the vinyl, followed by the removal of the glossy paper and pasting on FR-4 substrate (W/WO copper coated, Amazon). For the flexible substrate IR, the adhesive side was covered by another layer of vinyl or polyimide. For transferring the IR onto a conformal surface, the patterned metal was first transferred onto water-soluble tape, pasted on the desired surface by removal of the glossy paper, and finally released from the water-soluble tape in water. One layer of vinyl covering is then used to protect the bare copper traces.

Nutrient Sensor Fabrication: Detailed fabrication and characteristics of the nutrient sensors were presented in (*35*).

Pressure Sensor Fabrication: A patterned 1.5 cm spiral square copper electrode was pasted on a plastic cover slip and placed on a 3D printed box. After pouring Ecoflex-10 (Smooth On) layers of differing thickness on the top of this electrode, the top electrode (attached to plastic coverslip) was aligned on the Ecoflex. This setup was cured at room temperature for about four hours.

Temperature Sensor Fabrication: For the Smartcup pure Polyethylyne Glycol (PEG-1500, Alfa Aesar) solution is used, while for the wristband PEG-1500 is diluted in DI water at 1g/ml concentration. These were heated at 70 ºC and deposited on patterned 1 cm 2.25 turns spiral square copper electrodes. Top electrode layer was aligned and placed before the temperature could drop. Both electrodes were attached to a plastic coverslip. The completed sensor was embedded into an encapsulation layer to prevent the leaking of liquid PEG-1500. This was created by dipcoating the sensor in multiple layers of Ecoflex-50 (Smooth On).

pH Sensor Fabrication: A p(NIPAM-co-AA) hydrogel was used as interlayer synthesized by mixing 10% w/v N-Isopropylacrylamide (NIPAM, Sigma), 0.1% w/v methylene bisacrylamide (BIS, Sigma), 0.8% acrylic acid (AA, Sigma), 2.8% v/v N,N,N`,N`-Tetramethyl-ethylenediamine (TEMED, Sigma), and 0.28% w/v ammonium persulfate (APS, Sigma) at 0C. The precursor solution was deposited on 1 cm splitring, and the top splitring layer was aligned before final gelation. Sensor was equilibrated in pH 4 buffer solution for at

least 24 hours before experimentation. Detailed fabrication and characteristics of the nutrient sensors were presented in (*30*).

Salt Sensor Fabrication: Salt sensors are formed similar to pH sensors, however a PEGDA 700 hydrogel was used for interlayer instead. This was formed by mixing 10% v/v poly(ethylene glycol) diacrylate (PEGDA 700, Sigma), 0.2% v/v TEMED, and 0.1% w/v APS at 0C. Detailed fabrication and characteristics of the nutrient sensors were presented in (*30*).

Wristband Fabrication: A 3D mold made of PLA was used to form the base layer of the wristband (Ecoflex-30, Smooth On or PDMS, SYLGARDTM 184, base: curing agent = 10:1) with fixed compartments for each sensor. After placing four sensors (pressure, temperature, salt, pH) into their own compartment, another layer of (Ecpflex-30, PDMS-10:1) was deposited to form the wristband. Temperature and pressure sensors are completed sealed within the silicone, however salt and pH sensors possess openings in the bottom layer to enable sweat access.

In-Vitro Characterization: Networks were probed via either a nanoVNA (NanoRFE) or tabletopVNA (keysight, E5063A). Readout antenna is aligned against the network as noted in diagram, and spectral response of the network is probed.

Smartcup Testing: Four sensors (temperature alongside three nutrient sensors: salt-optimized, sugar-optimized, fat-optimized) were placed on the inside of the cup after fixing and aligning with the 5 cm diameter side of the IR. A 2.5 cm readout antenna was placed near the 3 cm diameter side of the IR to co-measure sensor response. Sensors were isolated in small compartments to comprehensively validate the lack of cross-coupling in individual sensor readout. First the temperature sensor was heated to 50 ℃ and measurements were taken while cooling slowly. Then, 100g/L glucose (D-Glucose, Sigma) was added to the sugar-optimized sensor, followed 20 uL oleic acid (Oleic Acid, Fisher Scientific) to the fat-optimized sensor, and finally 25 mg/dL salt (NaCl, Sigma) to the salt-optimized sensor. The total spectra was monitored throughout this process.

Wristband Testing: Manual pressure was applied on the pressure sensor, 10mg/dL NaCl was added to the salt sensor, deionized water was added to the pH sensor, while the temperature sensor was heated to 40ºC and allowed to cool while measurements were taken.

RF Circuit Simulations: For FR circuit simulation Keysight Pathwave Advanced Design System (ADS) was used for three sensors where synthetic R, L, and C are placed in parallel.

FDTD Simulations: The Finite-difference time-domain (FDTD) was adopted for EM simulations in the CST microwave studio simulations (RF module). A discreate port and open boundary conditions are used for a hexahedral mesh in the solution. A 1.5cm x 1.5cm SRR was coupled to the circular coil for CWOG and CWG. The interlayer of SRR has a thickness of 1mm and permittivity of 3. The distance between the sensor and circular antenna is 3mm. Frequency interval for the Gaussian shaped excitation function ranged from 0-1 GHz. All conducting plates are lossy pure copper with thickness of 50 micron unless otherwise stated. E and H fields were obtained at normalized maximum color plot.

In the case containing a curving Intermediate Relay (IR) to readout multiple sensors, the arc has a length of 40 mm and radian of 1 rad. The distance between antenna and IR, IR and sensor are both 1 mm. Three identical 1.1 cm x 1.1cm, 4.875-turns sensors (strip width and

gap are all 0.5 mm) were aligned along the axis of symmetry. The average magnetic field distribution is acquired by taking fields on the plane of symmetry.

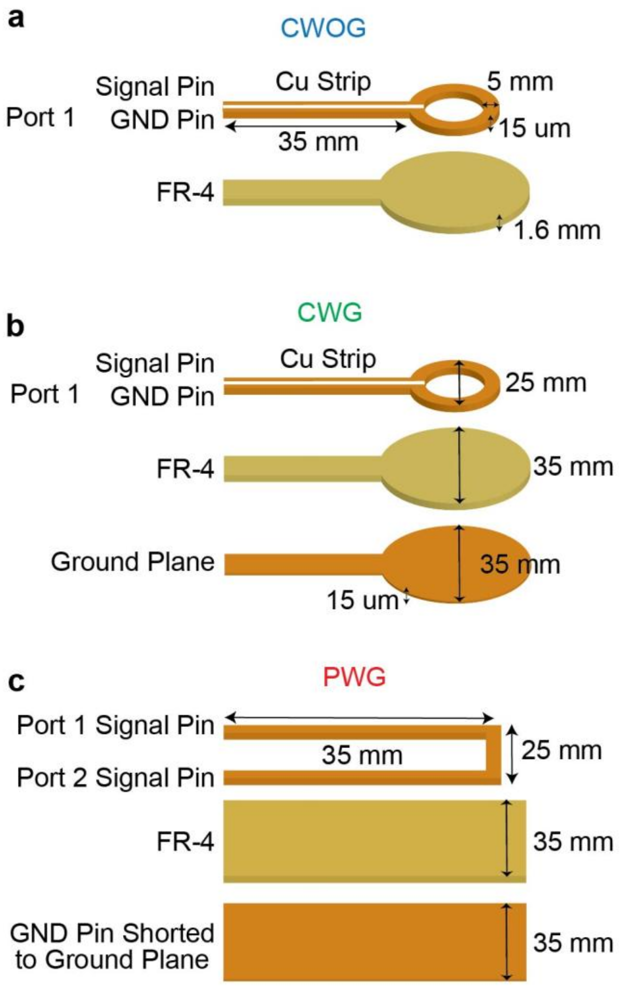

Supplementary Figure S1: **Readout Coil.** Geometry of (a) CWOG, (b) CWG, and (c) PWG readout antennas.

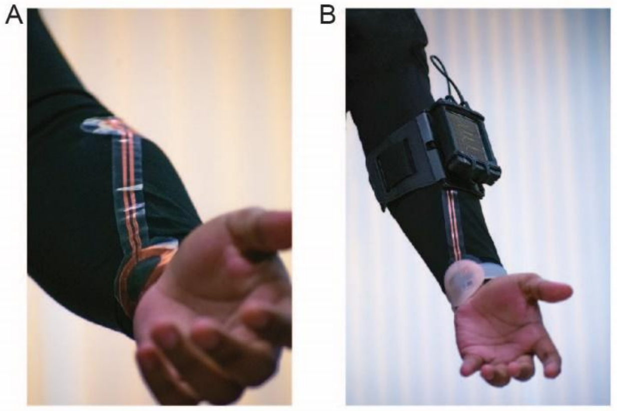

Supplementary Figure S2: IR integrated smart textile. (a) Flexible IR on the textile, (b) Placement of the Wristband and portable NanoVNA to read out multiparametric sensor state.

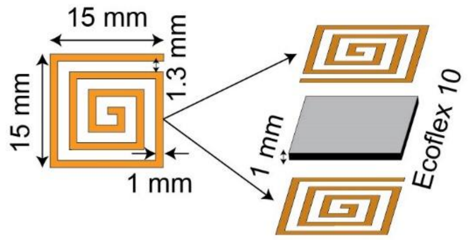

Supplementray Figure S3: A 3.25 turns spiral square trilayer sensor structure. Two spiral resonators were interceded by Ecoflex 10.

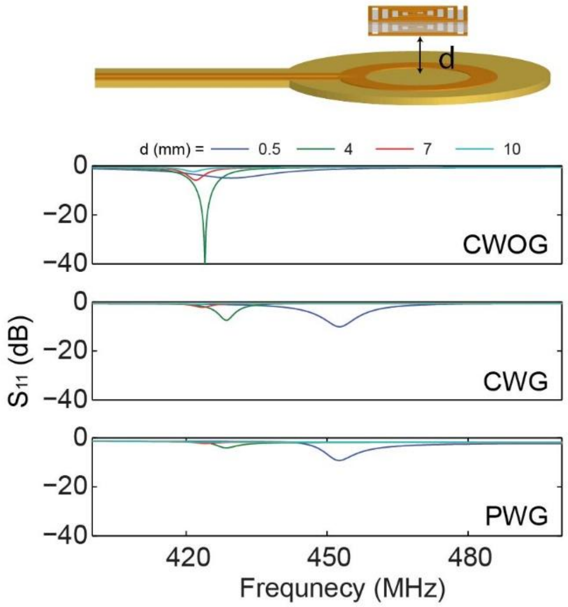

Supplementary Figure S4: **Effect of vertical distance between the sensor and the readout coil.** The sensor was placed on a 3D printed box (PLA material) and the box was clamped in two supports that moves vertically to change the distance. Change in the vertical distance modulates the coupling between the sensor and the readout coil, which in turn modulates the signal resonant frequency and the magnitude. Change in magnitude is higher in the CWOG, while change in resonant frequency is higher in CWG and PWG.

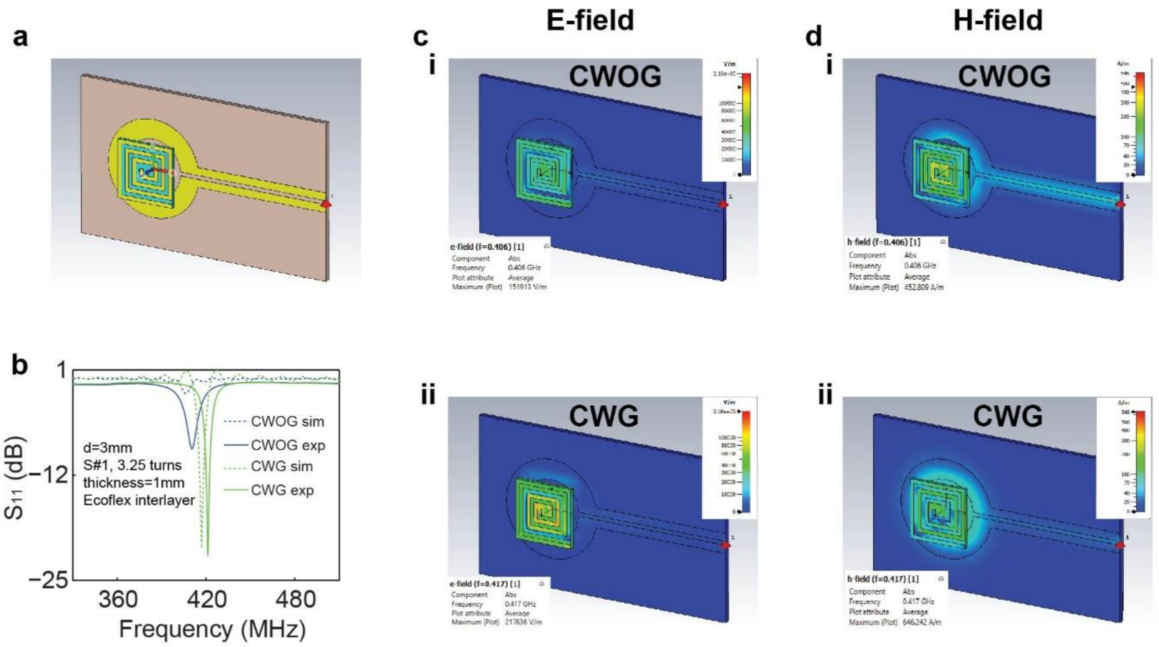

Supplementary Figure S5: Approximation of the sensor's operating region by FTDT simulation. (a) Geometry of the sensor and readout coil used in the simulation, (b) spectral response of the sensor by simulatsssion (dash line), and by experiment (solid line). (c) E-field and (d) H-field distribution of the sensor due to CWOG and CWG readout coils. Both E and H fileds are higher in magnitude at their resonance frequency for CWG than CWOG, which yields a higher Q in the S_{11} spectral response.

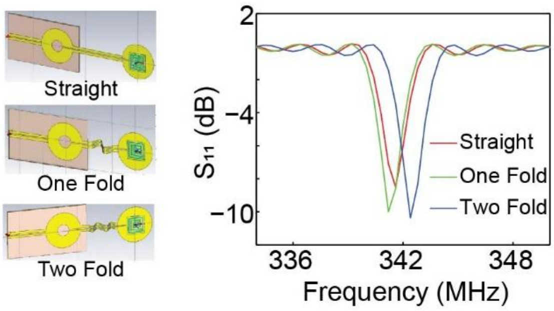

Supplementary Figure S6: Effect of bends in the IR on the measured spectral response of the sensor. Parameters for simulation: IR-antenna distance 0.5 mm, IR-sensor distance 1.1 mm, and IR length 110 mm.

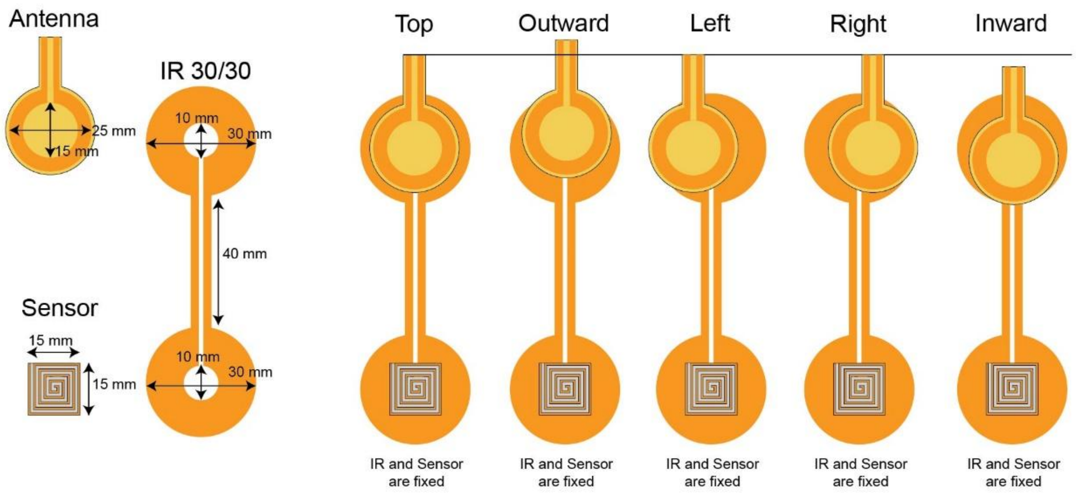

Supplementary Figure S7: Alignment between IR and antenna while sensor and IR placement is fixed. The width of the traces in the IR is larger than the width of the traces in the readout coil. This allows misalignment to be within the trace area of the IR.

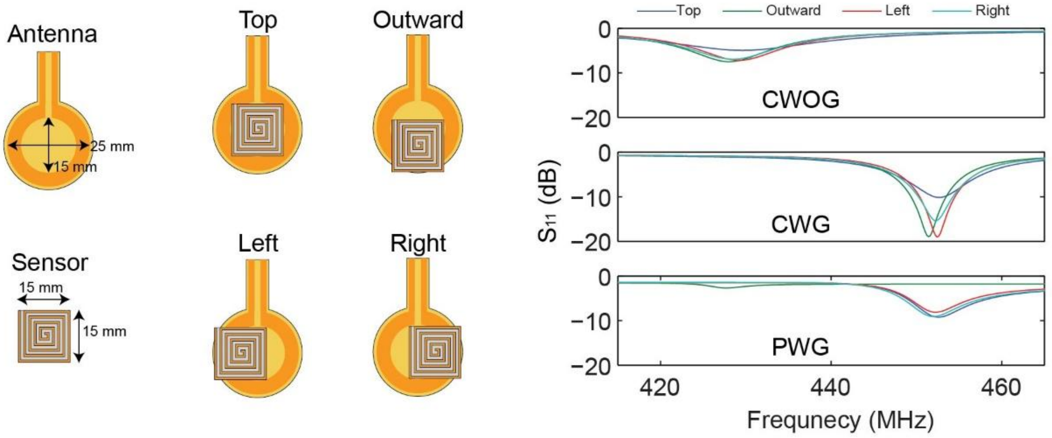

Supplementary Figure S8: **Effects of alignment between readout coil and the sensor without IR.** The measured resonant frequency modulates with changing alignment.

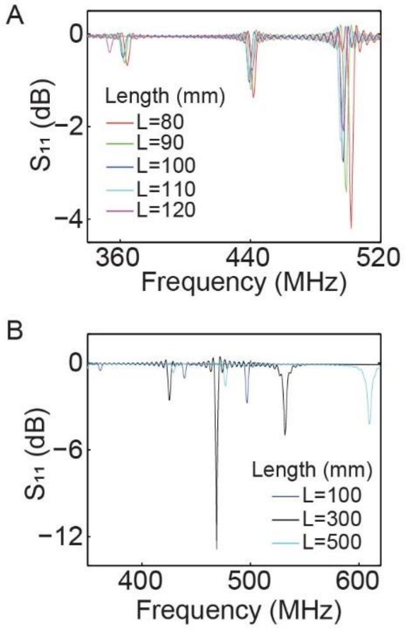

Supplementary Figure S9: Length Study. a) Effect of small changes in the length, b) large changes in the length. Parameters for simulation: IR-antenna distance 0.5 mm, and IR-sensor distance 1.1mm.

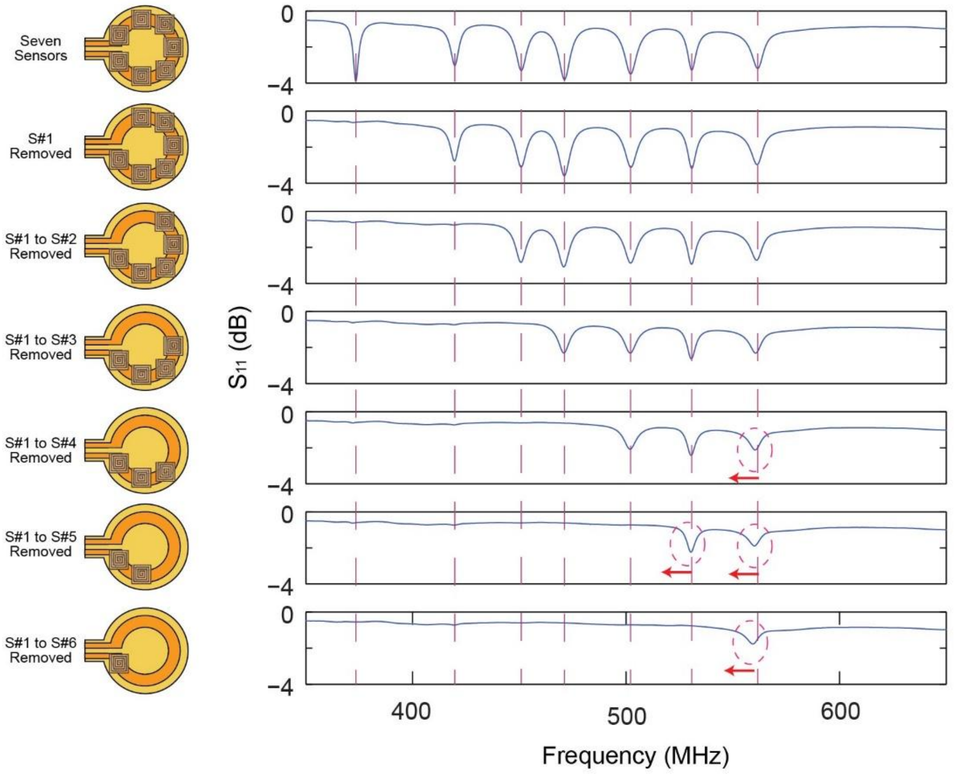

Supplementary Figure S10: **Positional coupling effect among sensors measured by one-by-one removal above a CWOG antenna.**

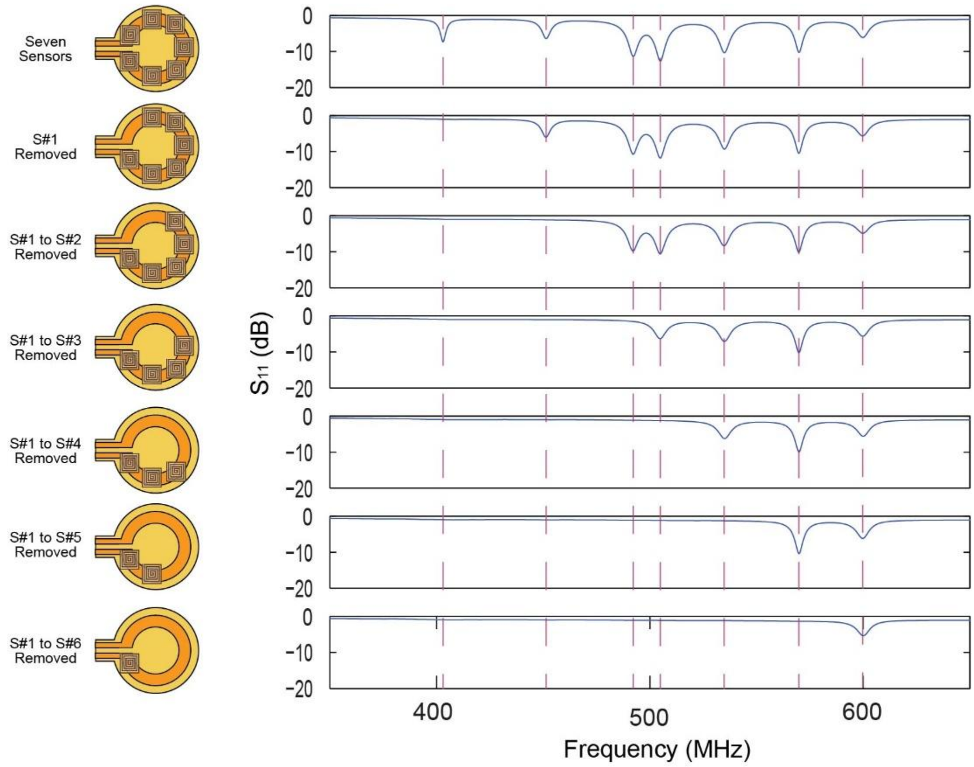

Supplementary Figure S11: **Positional coupling effect among sensors measured by one-by-one removal above a CWG antenna.**

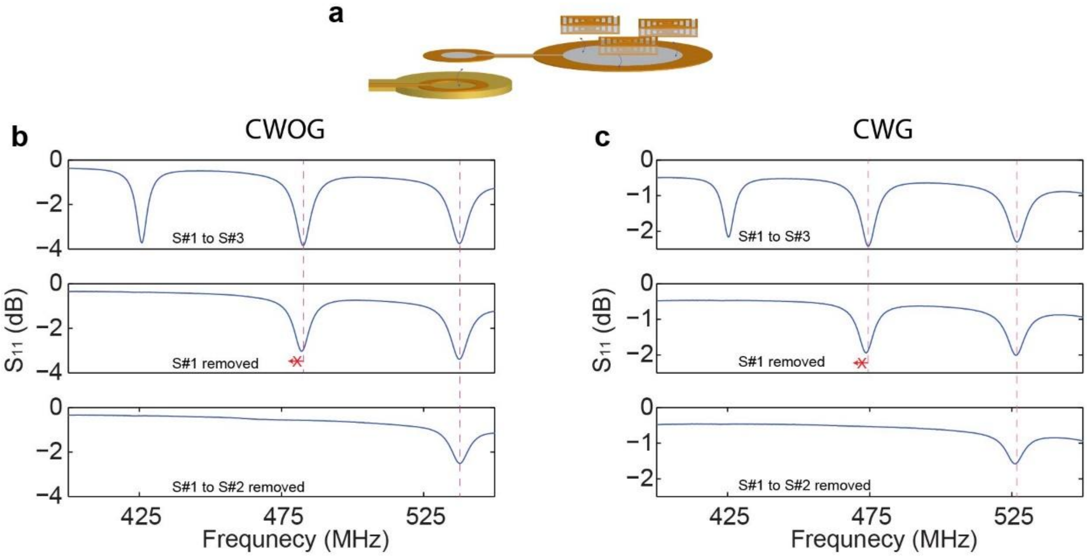

Supplementary Figure S12: **Position coupling effect among sensors with an IR30/50**. (a) Schematic of the readout coil, IR, and sensors, alongside network spectral response with (b) CWOG or (c) CWG. Both CWOG and CWG show cross coupling among sensors.

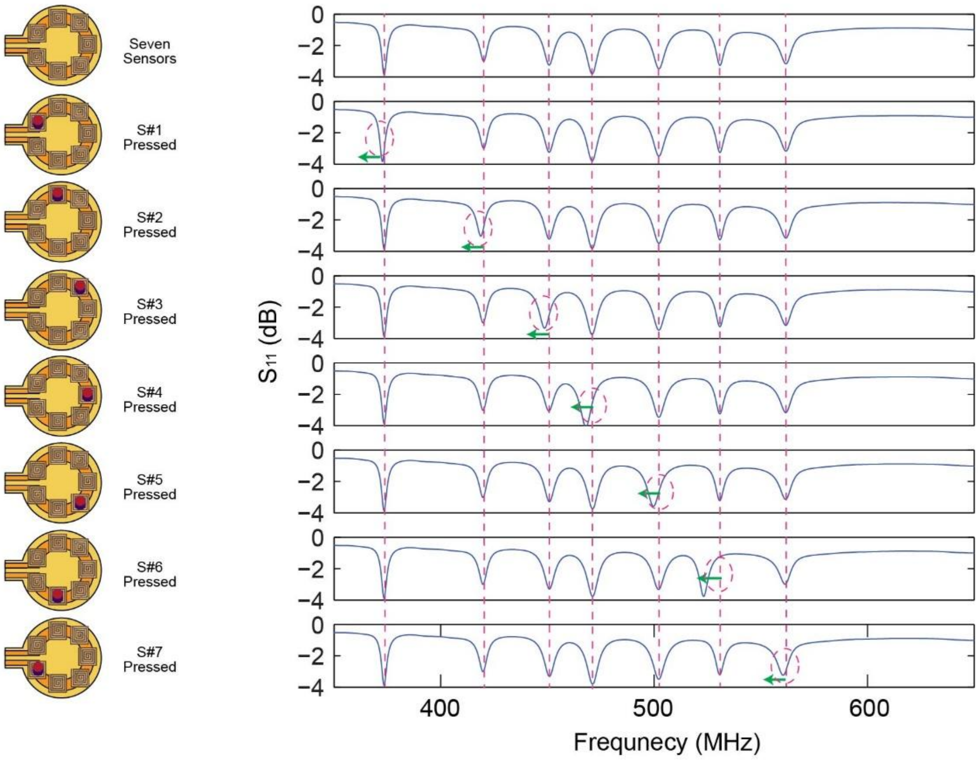

Supplementary Figure S13: Coupling among sensors due to individual sensor perturbation (pressure) in CWOG.

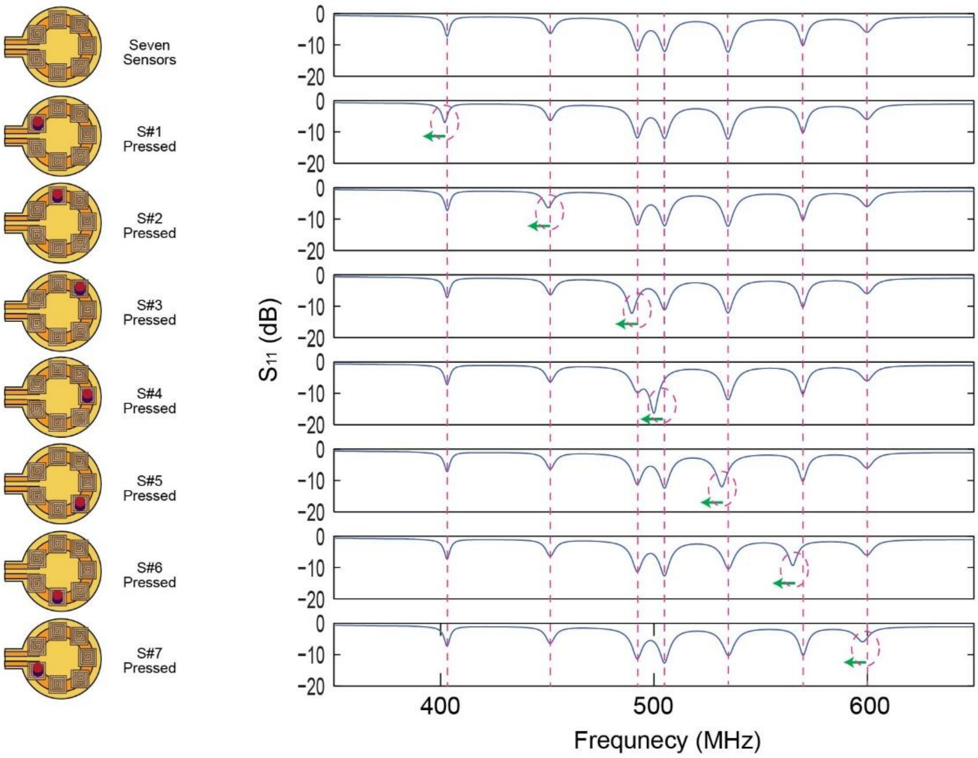

Supplementary Figure S14: Coupling among sensors due to individual sensor perturbation (pressure) in CWOG.

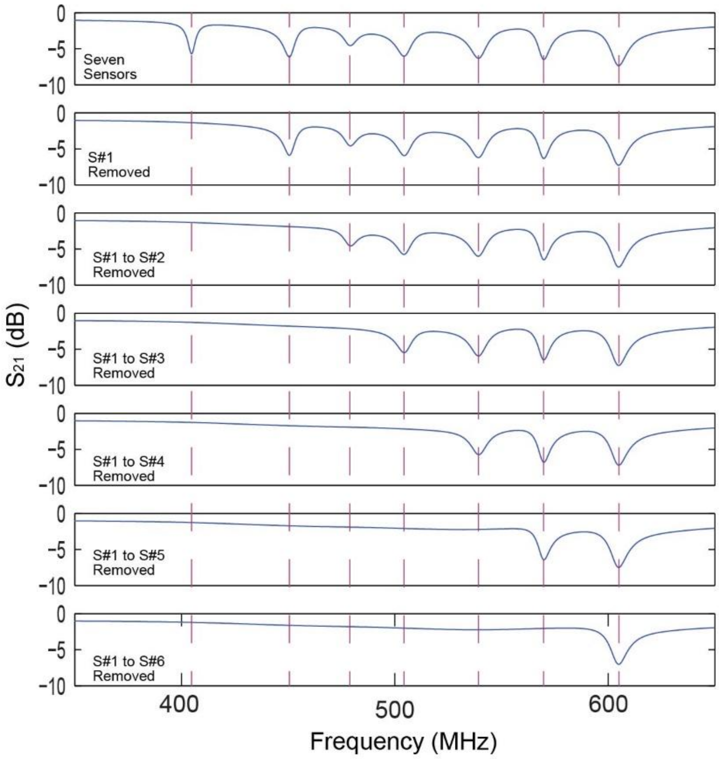

Supplementary Figure S15: **Positional coupling effect among sensors measured by one-by-one removal above a PWG antenna.**

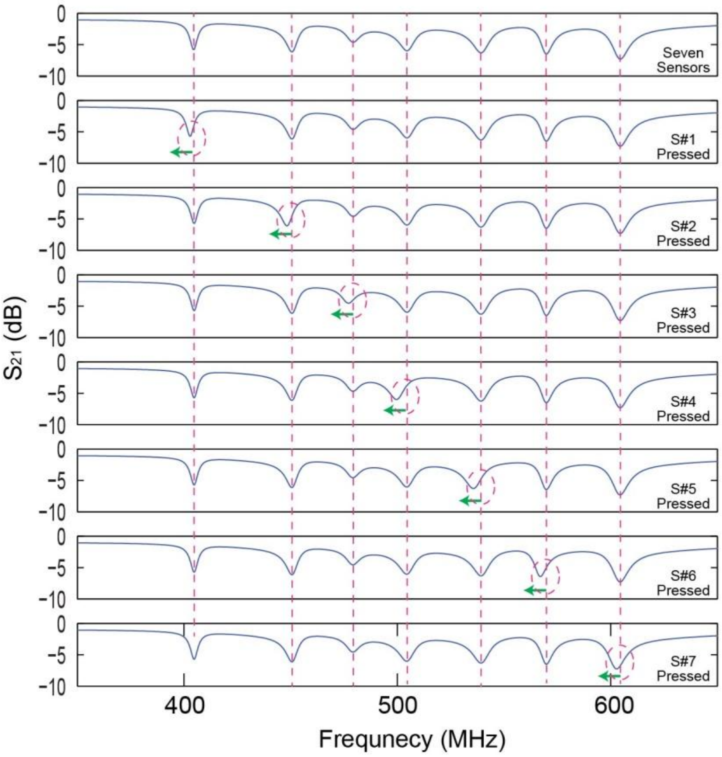

Supplementary Figure 16: **Coupling among sensors due to individual sensor perturbation (pressure) in PWG**.

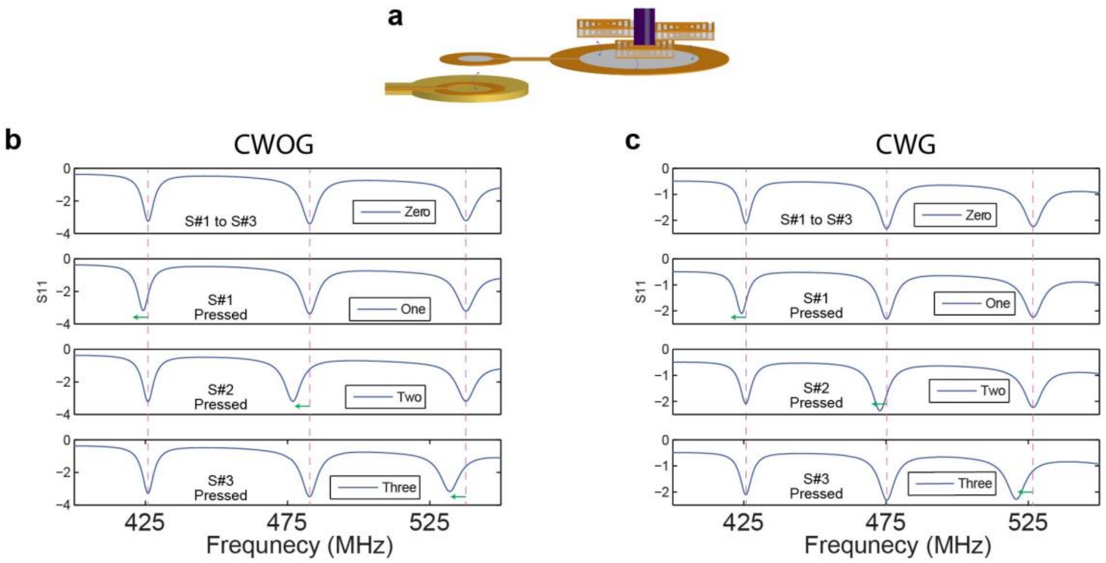

Supplementary Figure S17: Coupling among sensors due to individual sensor perturbation (pressure) with an interceding IR. (a) schematic of the placement of the readout coil, IR, and sensors, and network spectral response for (b) CWOG, or (c) CWG. The network exhibits no sensimetric coupling as long as the number of sensors stays static.

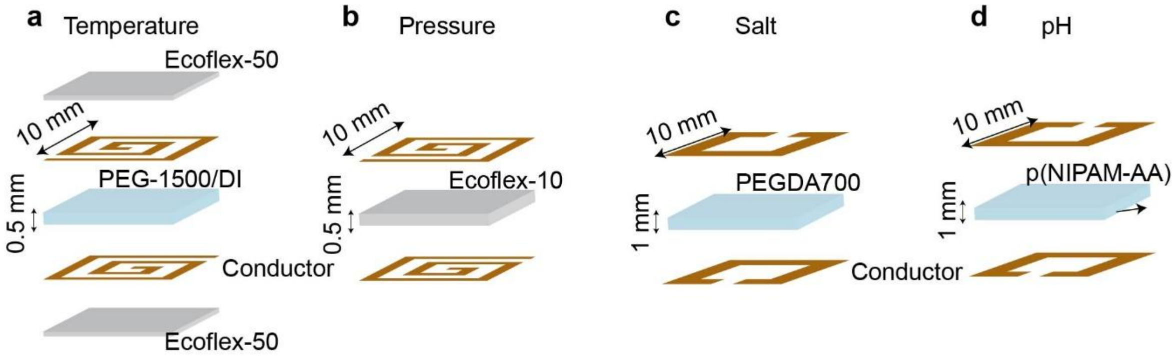

Supplementary Figure S18: Sensors used in wristband. (a) PEG-1500 interlayer temperature sensor, (b) Ecoflex-10 interlayer pressure sensor, (c) PEGDA700 hydrogel interlayer salt sensor, and (d) p(NIPAM-AA) hydrogel interlayer pH sensor.

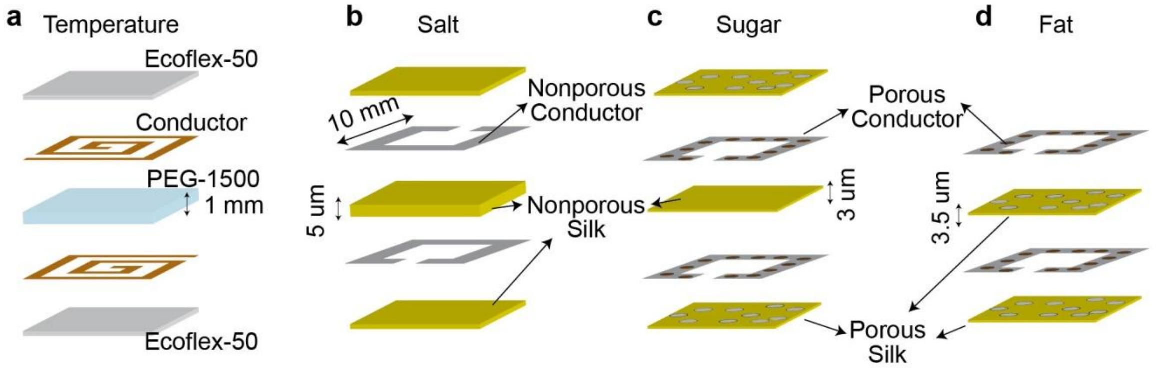

Supplementary Figure S19: **Sensors in SmartCup**. (a) PEG-1500 interlayer temperature sensor, (b) nonporous silk fibroin interlayer salt sensor, (c) nonporous silk fibroin interlayer sugar sensor, and (d) porous silk fibroin interlayer fat sensor.
